# Subclinical myocardial dysfunction assessed by cardiac magnetic resonance feature tracking predicts ventricular arrhythmias in early-stage hypertension

**DOI:** 10.3389/fcvm.2025.1632100

**Published:** 2025-10-30

**Authors:** Bin Fang, Weiwei Liao, Jianping Zhong, Junyuan Zhong

**Affiliations:** Medical Imaging Center, Ganzhou People's Hospital, Ganzhou Hospital-Nanfang Hospital, Ganzhou, China

**Keywords:** hypertension, cardiac dysfunction, CMR-FT, myocardial strain, ventricular arrhythmias

## Abstract

**Background:**

Substantial evidence supports the utility of cardiac magnetic resonance feature tracking (CMR-FT) in evaluating subclinical cardiac dysfunction. This study investigated the clinical value of CMR-derived left ventricular (LV) strain for detecting myocardial impairment in asymptomatic patients with hypertension (HTN) and explored its association with ventricular arrhythmias (VA).

**Methods:**

A retrospective analysis included 150 HTN patients [with/without LV hypertrophy (LVH)] and 60 healthy controls. Clinical data and CMR parameters were collected. Conventional LV functional indices and strain parameters—global longitudinal strain (GLS), global circumferential strain (GCS), and global radial strain (GRS)—were measured and compared across groups. The multivariable regression model was used to identify independent risk factors for VA.

**Results:**

Compared with controls, HTN patients showed significantly elevated LV mass index (LVMI) and maximal wall thickness (LVMWT) (*P* < 0.05). Both HTN subgroups (with/without LVH) exhibited impaired LV strain parameters (GLS, GCS, GRS) compared to controls (all *P* < 0.05). Systolic blood pressure (SBP), diastolic blood pressure (DBP), LVMI, and LVMWT correlated significantly with GRS, GCS, and GLS (*P* < 0.05), with LVMI demonstrating the strongest correlation with GLS (r = 0.58). Multivariable analysis identified GCS, BSA, and history of multi-drug antihypertensive therapy (Hx Multi-Drug AHT) as essential risk factors for VA in HTN patients. ROC analysis established GCS as the primary imaging predictor, with optimal VA discrimination at −17.005% (AUC = 0.848; sensitivity 69%, specificity 89.8%). The combined model (GCS + Hx Multi-Drug AHT + BSA) achieved superior performance (AUC = 0.923, 95% CI 0.868–0.960; sensitivity 85.71%, specificity 88.89%).

**Conclusion:**

CMR-FT-derived myocardial strain parameters demonstrate high sensitivity in detecting subclinical LV dysfunction in HTN patients. Furthermore, progressive impairment of GCS may serve as an independent risk factor for VA in this population. These findings provide imaging-based evidence to guide early interventions aimed at mitigating cardiac remodeling and arrhythmia development in HTN.

## Introduction

Hypertension (HTN), a critical global public health challenge affecting billions worldwide, continues to demonstrate suboptimal treatment and control rates (1). As the primary target organ, the heart develops characteristic pathological alterations including left ventricular hypertrophy (LVH), myocardial interstitial fibrosis, and progressive cardiac dysfunction (2). Notably, LVH and fibrosis induce increased ventricular myocyte excitability, abnormal electrical conduction, and regional relative ischemia-hypoxia, collectively elevating the risk of ventricular arrhythmias (VA) (3–5). VA may further exacerbate cardiac workload and promote myocardial remodeling (6). Substantial evidence confirms that early standardized antihypertensive therapy can reverse LVH progression and reduce VA incidence (7). It is crucial to early identify subclinical myocardial injury and predict VA risk.

Cardiac magnetic resonance (CMR) provides comprehensive assessment of cardiac morphology, function, and tissue characteristics. While conventional left ventricular (LV) functional parameters, such as left ventricular ejection fraction (LVEF), reflect global pump function, they often remain normal or even elevated during the HTN compensatory phase due to adaptive myocardial hypertrophy, making them insensitive for detecting early myocardial injury. The late gadolinium enhancement (LGE) technique accurately identifies focal replacement fibrosis (known as myocardial scarring) and serves as a key tool for detecting arrhythmogenic substrates (8, 9). However, in HTN patients, diffuse fibrosis predominates with LGE being rarely observed (10). Although extracellular volume (ECV) serves as a complementary marker for diffuse fibrosis, studies indicate that ECV changes are only mild and primarily observed in HTN patients with LVH (11). This suggests ECV may have limited predictive value for VA in early-stage HTN. Collectively, these limitations underscore the urgent need for more sensitive biomarkers to assess VA risk in early-stage HTN.

In recent years, cardiac magnetic resonance feature tracking (CMR-FT) has emerged as a novel noninvasive method for evaluating subclinical myocardial dysfunction by quantifying radial, circumferential, and longitudinal strain parameters (12). These strain metrics demonstrate significant correlations with myocardial hypertrophy severity, fibrotic distribution, and microcirculatory impairment (13, 14), establishing CMR-FT not only as a robust tool for detecting early cardiac dysfunction in HTN but also as a potential indicator of VA risk. Despite its promise, systematic studies on CMR-FT applications in HTN populations remain scarce, with limited exploration of its association with VA. This study employs CMR-FT to characterize subclinical LV mechanical properties in early-stage essential HTN and assess the correlation between strain parameters and VA in HTN patients.

## Materials and methods

### Clinical characteristics

**Figure 1:**
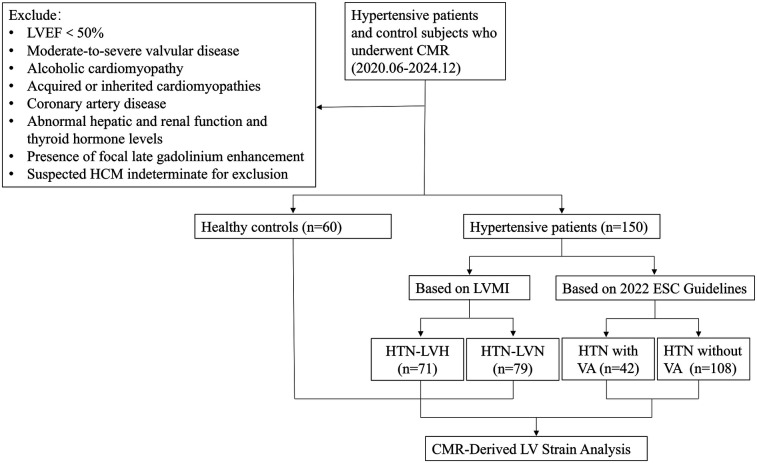
The flowchart of study participants.

This retrospective study was conducted with approval from the institutional review board, which waived the requirement for written informed consent (IRB no. MR-36-24-013077). We consecutively enrolled 150 consecutive patients with HTN who received treatment at our center between June 2020 and December 2024. Inclusion Criteria: (1) documented HTN [≥2 office measurements of systolic blood pressure (SBP) > 140 mmHg and/or diastolic blood pressure (DBP) > 90 mmHg or current antihypertensive therapy]; (2) LVEF ≥50% (derived from CMR diagnostic reports); (3) absence of secondary LVH etiologies (such as moderate-severe valvular disease, alcoholic cardiomyopathy, and acquired or inherited cardiomyopathies confirmed by clinical or paraclinical investigations); (4) no structural heart disease (coronary artery disease, rheumatic heart disease, etc.); (5) normal hepatic and renal function and thyroid hormone levels. Exclusion Criteria: (1) failure to meet inclusion criteria; (2) inadequate image quality for analysis; (3) presence of focal LGE; (4) suspected hypertrophic cardiomyopathy (HCM) with diffuse ventricular wall thickening indistinguishable from HTN. Figure 1 shows the flow chart of the study.

HTN patients were stratified into two groups based on left ventricular mass index (LVMI): HTN-LVH group (*n* = 71, 54 males, mean age 51.03 ± 12.50 years) and HTN-LVN group (*n* = 79, 50 males, mean age 56.75 ± 11.38 years). LVH was defined as LVMI >81 g/m² for men and >61 g/m² for women (15). A healthy control group matched by age and sex (*n* = 60, 29 males; mean age 50.42 ± 12.84 years) was concurrently enrolled, with confirmed normal cardiac structure/function and absence of HTN or other cardiovascular diseases. Patients with arrhythmia on routine electrocardiogram (ECG) or recurrent arrhythmias that required follow-up underwent 24-hour dynamic electrocardiography (DCG) monitoring. DCG monitoring identified 42 HTN patients with VA (HTN-LVH:23, HTN-LVN:19). VA were classified per the 2022 ESC Guidelines as: non-sustained ventricular tachycardia (≥3 consecutive premature ventricular contractions, rate >100 beats per minute, duration ≤30 s), sustained ventricular tachycardia (>30 s or requiring urgent intervention), or ventricular fibrillation (9). Clinical data for all participants were systematically collected.

### CMR imaging protocol

All examinations were performed on a 3.0 T MRI scanner (Skyra, Siemens Healthineers, Germany). Using an 18-channel body matrix coil with retrospective ECG gating and respiratory compensation. Cine imaging was acquired using balanced steady-state free precession (b-SSFP) sequence with the following parameters: repetition time (TR) 39.48 ms, echo time (TE) 1.43 ms, flip angle 47°, field of view (FOV) 340 × 285 mm², slice thickness 5 mm with no gap. Imaging planes included two-chamber, three-chamber, four-chamber views, and short-axis stacks covering the LV from mitral annulus to apex. For LGE imaging, Gd-DTPA (Beijing Beilu Pharmaceutical Corporation, China) was administered intravenously at 0.05 mmol/kg followed by 20 mL saline flush. LGE images were obtained 8–10 min post-injection using a 2D phase-sensitive inversion recovery sequence. The relevant parameters are as follows: TR 750 ms, TE 2.1 ms, flip angle 20°, FOV 350 × 262.5 mm², slice thickness 5 mm with no gap, and inversion time (TI) individually adjusted between 260 and 370 ms.

### Image analysis

Conventional cardiac function parameters were analyzed using the Syngo.via post-processing workstation (Siemens Healthineers, Germany). All cine images were imported, and the software automatically delineated endocardial and epicardial contours at end-systole and end-diastole. Following manual adjustment to exclude papillary muscles and trabeculae, parameters were normalized to body surface area (BSA). The derived indices included: LVEF, LVMI, left ventricular end-diastolic volume index (LVEDVI), left ventricular end-systolic volume index (LVESVI), cardiac index (CI). Maximum left ventricular wall thickness (LVMWT) was measured at end-diastole from short-axis cine images. LGE images were independently evaluated by two experienced radiologists to identify focal fibrotic lesions.

**Figure 2:**
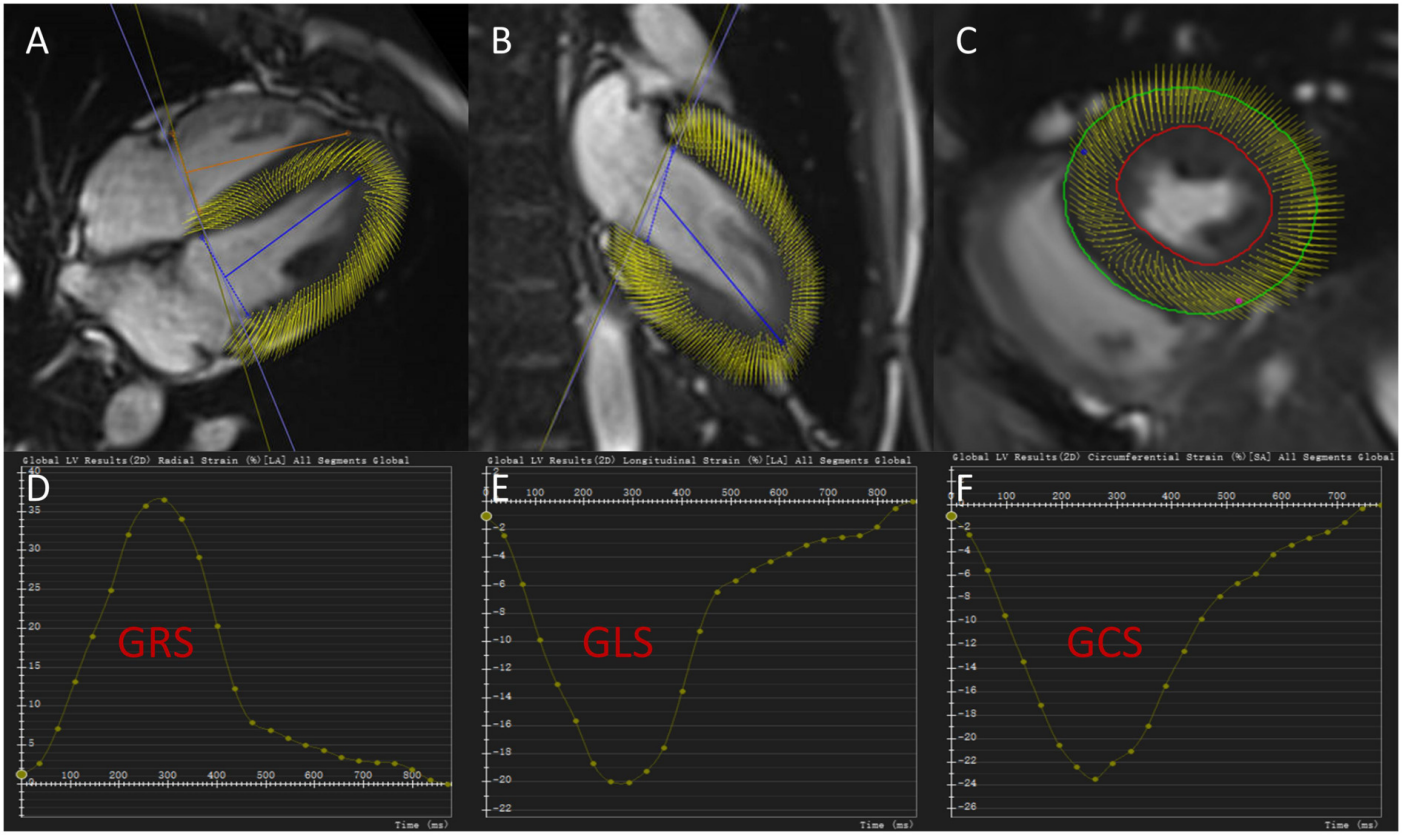
Cardiac magnetic resonance tissue tracking in four-chamber, two-chamber long-axis, and short-axis cine views at end-diastole **(A–C)**. Global radial strain curve, global longitudinal strain curve and global circumferential strain curve for a hypertensive patient without left ventricular hypertrophy **(D–F)**. GRS, global radial strain; GCS, global circumferential strain; GLS, global longitudinal strain.

LV myocardial strain measurements were performed using CVI42 (version 5.11.1, Circle Cardiovascular Imaging, Calgary, Canada). Following import of the cine sequences, endocardial and epicardial contours at end-diastole were automatically identified by the software. After manual correction, the following strain and strain rate parameters were calculated: global longitudinal strain (GLS), global radial strain (GRS), global circumferential strain (GCS), global peak systolic longitudinal strain rate (sGLSR), systolic radial strain rate (sGRSR), systolic circumferential strain rate (sGCSR), global peak early-diastolic longitudinal strain rate (eGLSR), early-diastolic radial strain rate (eGRSR), early-diastolic circumferential strain rate (eGCSR) (are shown in Figure 2).

### Reproducibility of strain parameters

To evaluate measurement reliability, we randomly selected CMR images from 20 HTN patients and 10 healthy controls for reproducibility validation. Two radiologists, each with over 2 years of experience in CMR post-processing, independently measured LV strain parameters to assess inter-observer agreement. Intra-observer reproducibility was determined by selecting the same radiologist repeat measurements on the same 30 subjects after a 3-month interval.

### Statistical analysis

Continuous variables were tested for normality with the Kolmogorov–Smirnov test and expressed as mean ± standard deviation (normally distributed) or median (interquartile range) (non-normal). Categorical variables were reported as frequencies (%). For three-group comparisons, normally distributed data were analyzed by one-way analysis of variance (ANOVA) with Bonferroni *post-hoc* correction, and non-normally distributed data by the Kruskal–Wallis H test. HTN patients with and without VA were compared using independent *t*-tests (normal data) or Mann–Whitney *U*-tests (non-normal data). Categorical variables were analyzed with chi-square or Fisher's exact tests. Pearson correlation evaluated associations between strain parameters and other continuous variables. Variable selection was performed using least absolute shrinkage and selection operator (LASSO) regression, with subsequent multivariate logistic regression identifying independent risk factors for VA in HTN patients. Receiver operating characteristic (ROC) curves assessed the discriminative ability of strain parameters for VA in HTN patients. Intraclass correlation coefficients (ICC) quantified intra- and inter-observer reliability of LV strain measurements. LASSO regression was performed using the glmnet package (version 4.18) in R, with all other analyses conducted in SPSS 25.0 (IBM Corp., NY). A two-tailed *P* < 0.05 defined statistical significance.

## Results

### Comparison of clinical characteristics and conventional LV function parameters

Comparisons of clinical characteristics and conventional LV function parameters among the control, HTN-LVN, and HTN-LVH groups are summarized in Table 1. Compared with the control group, HTN patients (HTN-LVN and HTN-LVH) had significantly higher body weight, DBP, and SBP (all *P* < 0.05). Smoking prevalence was higher in the HTN-LVH group than in controls (*P* < 0.05). Notably, LVEDVI demonstrated an initial decrease followed by an increase across the control, HTN-LVN, and HTN-LVH groups (*P* < 0.05), aligning with hypertensive LV remodeling. Conversely, LVMI and LVMWT showed a progressive increase with statistically significant differences across all subgroups (all *P* < 0.05). Age, sex, height, alcohol use, BSA, heart rate (HR), stroke volume index (SVI), CI, and LVEF did not differ significantly among the groups. Furthermore, in comparisons between the two HTN subgroups, the HTN-LVH group exhibited significantly higher SBP and a greater proportion of patients with a history of multi-drug antihypertensive therapy (Hx Multi-Drug AHT) (both *P* < 0.05), while no significant differences were observed in HTN duration, diabetes prevalence, or hyperlipidemia prevalence (despite 3 missing hyperlipidemia cases, sensitivity analysis classifying missing data as “Unverified” confirmed result robustness: Δ*P* < 0.10 vs. primary analysis; Supplementary Table 1).

**Table 1:** Comparison of clinical characteristics and conventional cardiac function parameters between HTN-LVH group, HTN-LVN group and control group.

Parameters	Controls (60)	HTN-LVN (79)	HTN-LVH (71)	*P* *	*P* #	*P* $
Age (years)	52.25 ± 11.79	56.20 ± 10.64	51.45 ± 12.29	0.071	1.000	0.076
Man, *n* (%)	29 (18%)	50 (63.3%)	49 (69.0%)	0.078	0.066	0.393
Smoking, *n* (%)	12 (20.0%)	22 (27.8%)	29 (40.8%)	0.286	0.010	0.093
Alcohol consumption, *n* (%)	9 (15.0%)	14 (17.7%)	17 (23.9%)	0.669	0.201	0.347
Body weight (kg)	62.38 ± 10.02	69.50 ± 11.69	70.61 ± 12.18	0.001	<0.001	1.000
Height (cm)	162 (159, 168)	168 (157, 171)	165 (160, 170)	0.798	1.000	1.000
BSA (m^2^)	1.66 ± 0.19	1.73 ± 0.24	1.74 ± 0.25	0.605	0.841	0.962
HR (beats per minute)	75.5 (70.1, 88.2)	73.3 (64.2, 81.5)	71.4 (63.4, 79.3)	0.480	0.209	0.944
DBP (mmHg)	116.05 ± 8.63	152.01 ± 15.53	161.06 ± 16.13	<0.001	<0.001	0.081
SBP (mmHg)	73.55 ± 8.18	88.52 ± 12.54	93.70 ± 15.75	<0.001	<0.001	0.013
LVEDVI (mL/m^2^)	68.60 ± 10.38	62.82 ± 14.41	69.88 ± 14.36	0.037	1.000	0.004
LVESVI (mL/m^2^)	25.77 ± 6.06	22.76 ± 7.23	25.08 ± 11.14	0.025	0.958	0.358
SVI (mL/m^2^)	42.93 ± 7.19	39.49 ± 9.68	44.30 ± 11.26	0.116	1.000	0.008
CI (L/min/m^2^)	2.99 (2.51, 3.30)	2.90 (2.38, 3.36)	3.14 (2.63, 3.52)	0.895	0.600	0.160
LVEF (%)	63.06 ± 5.58	63.99 ± 6.78	65.11 ± 8.87	1.000	0.328	1.000
LVMI (g/m^2^)	57.86 ± 12.68	68.54 ± 12.39	99.62 ± 22.76	<0.001	<0.001	<0.001
LVMWT (mm)	9.04 ± 1.54	11.98 ± 2.27	14.79 ± 3.17	<0.001	<0.001	<0.001
Diabetes, *n* (%)	─	14 (17.7%)	11 (15.7%)	─	─	0.743
Hyperlipidemia, *n* (%)^a^	─	31 (40.3%)	38 (54.3%)	─	─	0.128
History of HTN ≥ 5 years, *n* (%)	─	32 (40.5%)	34 (47.9%)	─	─	0.363
Hx Multi-Drug AHT, *n* (%)	─	21 (26.6%)	31 (43.7%)	─	─	0.028
Users of Statins, *n* (%)	─	44 (55.7%)	46 (64.8%)	─	─	0.256

Controls, Healthy controls; HTN-LVN, hypertensive patients without left ventricular hypertrophy; HTN-LVH, hypertensive patients with left ventricular hypertrophy; BSA, body surface area; HR, heart rate; DBP, diastolic blood pressure; SBP, systolic blood pressure; LVEDVI, left ventricular end-diastolic volume index; LVESVI, left ventricular end-systolic volume index; SVI, stroke volume index; CI, cardiac index; LVMI, left ventricular mass index; LVMWT, maximum left ventricular wall thickness; Hx Multi-Drug AHT, History of multi-drug antihypertensive therapy (concurrent use of ≥2 drug classes [e.g., Beta-blocker + Calcium blocker] for ≥3 months.

^a^ Excluded unverified hyperlipidemia; denominators = verified cases: HTN-LVN:77/79, HTN-LVH:70/71.

* Comparison of the control group and HTN-LVN group.

# Comparison of the control group and HTN-LVH group.

$ Comparison of the HTN-LVN group and HTN-LVH group.

### Comparison of LV strain parameters

**Figure 3:**
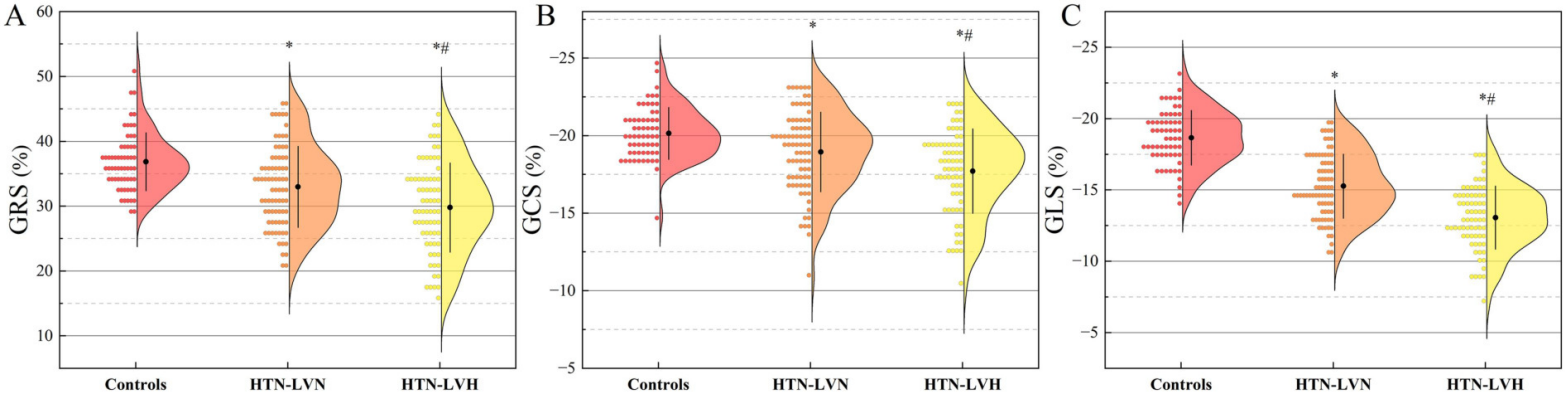
Half-violin plots displaying left ventricular strain parameters for the three study groups: global radial strain (GRS, **A**), global circumferential strain (GCS, **B**), and global longitudinal strain (GLS, **C**). Positive and negative values indicate the direction of myocardial motion. HTN-LVN, hypertensive patients without left ventricular hypertrophy; HTN-LVH, hypertensive patients with left ventricular hypertrophy. *, *P* < 0.05 vs. Controls; #, *P* < 0.05 vs. HTN-LVN.

The LV strain parameters among control, HTN-LVN, and HTN-LVH groups are presented in Table 2 and Figure 3. GRS, GCS, GLS, and strain rate parameters (eGRSR, eGCSR, eGLSR) exhibited a progressive decrease across groups, with statistically significant differences observed in all intergroup comparisons (all *P* < 0.05). However, sGRSR and sGCSR were significantly reduced in the HTN-LVH group compared to controls (both *P* < 0.05), while no significant differences were observed between other subgroups (all *P* > 0.05).

**Table 2:** Comparison of myocardial strain parameters between control group, HTN-LVN group and HTN-LVH group.

Parameters	Controls (60)	HTN-LVN (79)	HTN-LVH (71)	*P* *	*P* #	*P* $
GRS (%)	36.86 ± 4.48	32.98 ± 6.24	29.80 ± 6.87	<0.001	<0.001	0.011
GCS (%)	−20.15 ± 1.67	−18.95 ± 2.56	−17.72 ± 2.71	0.003	<0.001	0.015
GLS (%)	−18.66 ± 1.92	−15.27 ± 2.24	−12.66 ± 3.91	<0.001	<0.001	<0.001
sGRSR (s^−1^)	1.93 ± 0.39	1.82 ± 0.44	1.67 ± 0.43	0.477	0.002	0.081
sGCSR (s^−1^)	−1.07 (−1.23, −0.97)	−1.03 (−1.21, −0.94)	−0.99 (−1.15, −0.91)	0.104	0.010	0.290
sGLSR (s^−1^)	−0.95 (−1.04, −0.90)	−0.92 (−1.03, −0.82)	−0.81 (−0.93, −0.73)	0.028	<0.001	0.001
eGRSR (s^−1^)	−1.94 ± 0.43	−1.47 ± 0.50	−1.31 ± 0.39	<0.001	<0.001	<0.001
eGCSR (s^−1^)	1.01 ± 0.17	0.87 ± 0.17	0.78 ± 0.17	<0.001	<0.001	<0.001
eGLSR (s^−1^)	1.03 ± 0.20	0.85 ± 0.15	0.73 ± 0.15	<0.001	<0.001	<0.001

Controls, Healthy controls; HTN-LVN, hypertensive patients without left ventricular hypertrophy; HTN-LVH, hypertensive patients with left ventricular hypertrophy; GRS, global radial strain; GCS, global circumferential strain; GLS, global longitudinal strain; sGRSR, global peak systolic radial strain rate; sGCSR, global peak systolic circumferential strain rate; sGLSR, global peak systolic longitudinal strain rate; eGRSR, global peak early-diastolic radial strain rate; eGCSR, global peak early-diastolic circumferential strain rate; eGLSR, global peak early-diastolic longitudinal strain rate.

* Comparison of the control group and HTN-LVN group.

# Comparison of the control group and HTN-LVH group.

$ Comparison of the HTN-LVN group and HTN-LVH group.

### Correlation analysis

**Figure 4:**
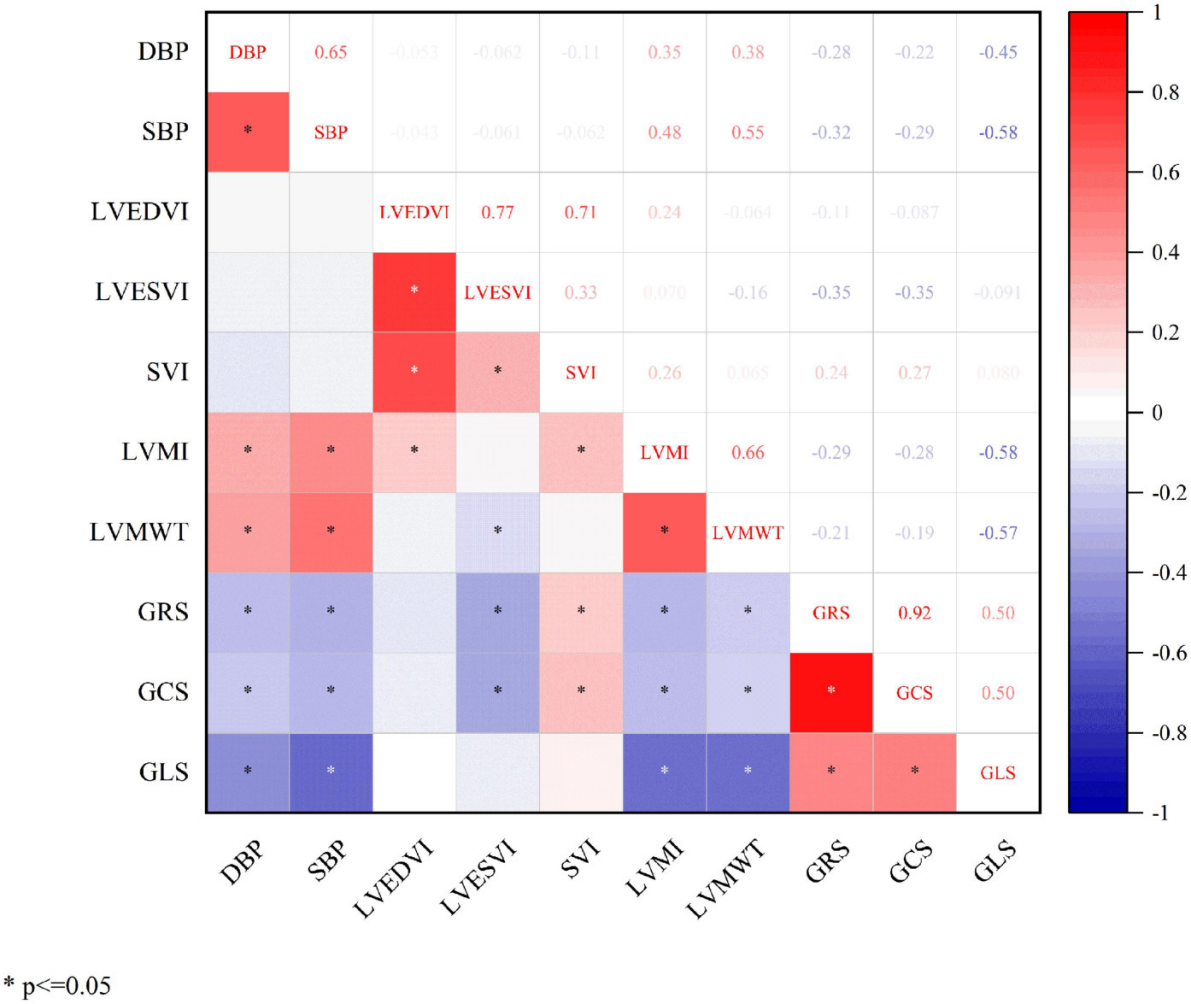
Correlation analysis between global left ventricular myocardial strain and basic functional parameters. DBP, diastolic blood pressure; SBP, systolic blood pressure; LVEDVI, left ventricular end-diastolic volume index; LVESVI, left ventricular end-systolic volume index; SVI, stroke volume index; LVMI, left ventricular mass index; LVMWT, maximum left ventricular wall thickness; GRS, global radial strain; GCS, global circumferential strain; GLS, global longitudinal strain.

Pearson correlation analysis revealed significant linear relationships between LV global strain parameters and cardiac functional indices in all participants (Figure 4). DBP showed inverse correlations with GRS (r = −0.28), GCS (r = −0.22), and GLS (r = −0.45). Similarly, SBP correlated negatively with GRS (r = −0.32), GCS (r = −0.29), and GLS (r = −0.58). LVESVI demonstrated negative correlations with GRS (r = −0.35) and GCS (r = −0.35), while SVI showed positive correlations with GRS (r = 0.24) and GCS (r = 0.27). LVMI correlated inversely with GRS (r = −0.29), GCS (r = −0.28), and GLS (r = −0.58). LVMWT exhibited negative correlations with GRS (r = −0.21), GCS (r = −0.19), and GLS (r = −0.57). All correlations were statistically significant (*P* < 0.05).

### Comparison of parameters between HTN patients with and without VA

As shown in Table 3, HTN patients with VA demonstrated significantly higher LVEDVI and LVESVI, lower LVEF, and a higher proportion of Hx Multi-Drug AHT compared to those without VA (all *P* < 0.05). No significant differences were observed between the two groups in terms of height, weight, age, sex distribution, LVMI, LVMWT, SVI, CI, or HR. Notably, patients with VA exhibited reduced myocardial strain parameters including GRS, GCS, GLS, sGRSR, sGLSR, eGRSR, eGCSR, and eGLSR compared to VA-free patients (all *P* < 0.05), with the exception of sGCSR which showed no significant difference.

**Table 3:** Parameter comparison of HTN patients without VA and with VA.

Parameters	HTN without VA (108)	HTN with VA (42)	*P*
Age (years)	53.81 ± 12.18	54.62 ± 12.46	0.719
Man, *n* (%)	73 (67.59%)	31 (73.81%)	0.458
BSA (m^2^)	1.79 ± 0.19	1.58 ± 0.28	<0.001
HR (beats per minute)	73.61 ± 13.10	75.93 ± 18.69	0.465
SBP (mmHg)	154.55 ± 16.01	158.21 ± 17.48	0.373
DBP (mmHg)	90.72 ± 13.87	91.62 ± 15.65	0.732
Diabetes, *n* (%)	15 (14.0%)	10 (23.8%)	0.150
Hyperlipidemia, *n* (%)^a^	47 (45.2%)	22 (55.0%)	0.403
History of HTN ≥ 5 years, *n* (%)	44 (40.7%)	22 (52.4%)	0.197
Hx Multi-Drug AHT, *n* (%)	26 (24.1%)	26 (61.9%)	<0.001
Users of Statins, *n* (%)	60 (55.6%)	30 (71.4%)	0.075
LVEDVI (mL/m^2^)	63.63 ± 13.88	72.68 ± 15.15	<0.001
LVESVI (mL/m^2^)	21.71 ± 7.21	29.38 ± 11.71	<0.001
SVI (mL/m^2^)	41.36 ± 9.25	42.82 ± 13.81	0.453
CI (L/min/m^2^)	3.00 ± 0.66	3.12 ± 1.05	0.501
LVEF (%)	66.03 ± 6.99	60.64 ± 8.59	<0.001
LVMI (g/m^2^)	82.69 ± 24.05	84.68 ± 23.26	0.647
LVMWT (mm)	13.26 ± 2.98	13.42 ± 3.32	0.776
GRS (%)	33.55 ± 5.89	26.13 ± 5.73	<0.001
GCS (%)	−19.32 ± 2.04	−15.62 ± 2.66	<0.001
GLS (%)	−14.48 ± 3.70	−12.89 ± 2.07	0.009
sGRSR (s^−1^)	1.87 ± 0.41	1.45 ± 0.36	<0.001
sGCSR (s^−1^)	−1.05 (−1.22, −0.95)	−0.95 (−1.00, −0.81)	0.067
sGLSR (s^−1^)	−0.90 (−1.02, −0.80)	−0.79 (−0.88, −0.69)	<0.001
eGRSR (s^−1^)	−1.52 ± 0.39	−1.08 ± 0.46	<0.001
eGCSR (s^−1^)	0.85 ± 0.17	0.76 ± 0.19	0.004
eGLSR (s^−1^)	0.81 ± 0.15	0.74 ± 0.18	0.021

HTN, hypertension; VA, ventricular arrhythmia; BSA, body surface area; HR, heart rate; SBP, systolic blood pressure; DBP, diastolic blood pressure; Hx Multi-Drug AHT, History of multi-drug antihypertensive therapy (concurrent use of ≥2 drug classes [e.g., Beta-blocker + Calcium blocker] for ≥3 months; LVEDVI, left ventricular end-diastolic volume index; LVESVI, left ventricular end-systolic volume index; SVI, stroke volume index; CI, cardiac index; LVMI, left ventricular mass index; LVMWT, maximum left ventricular wall thickness; GRS, global radial strain; GCS, global circumferential strain; GLS, global longitudinal strain; sGRSR, global peak systolic radial strain rate; sGCSR, global peak systolic circumferential strain rate; sGLSR, global peak systolic longitudinal strain rate; eGRSR, global peak early-diastolic radial strain rate; eGCSR, global peak early-diastolic circumferential strain rate; eGLSR, global peak early-diastolic longitudinal strain rate.

^a^ Excluded unverified hyperlipidemia; denominators = verified cases: HTN without VA: 105/108, HTN with VA: 42/42.

### Risk factor analysis and diagnostic performance of strain parameters for VA in HTN patients

**Figure 5:**
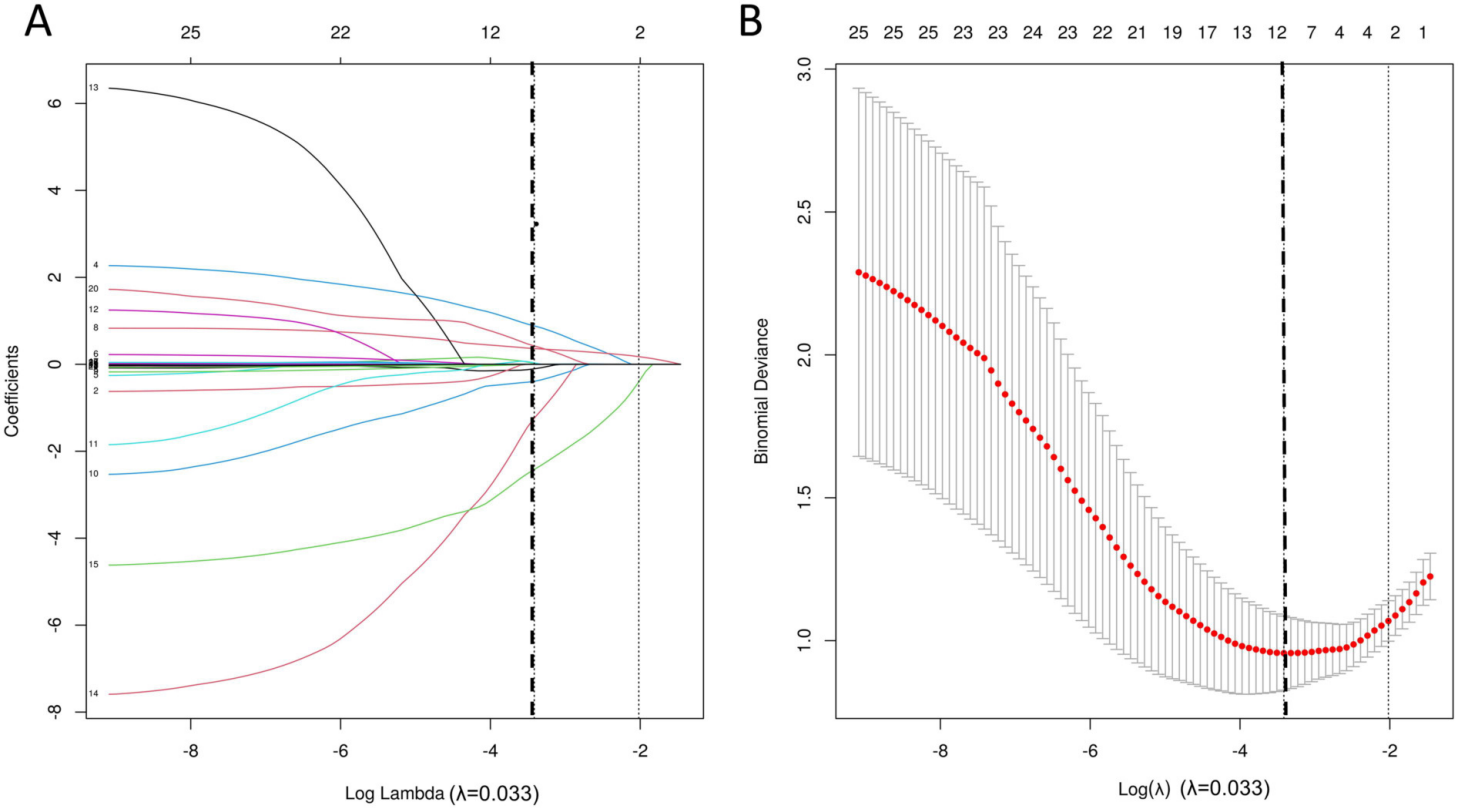
Variable selection process using LASSO regression. **(A)** Coefficient trajectories of candidate predictors; **(B)** Cross-validation error curve with optimal lambda (*λ*).

**Figure 6:**
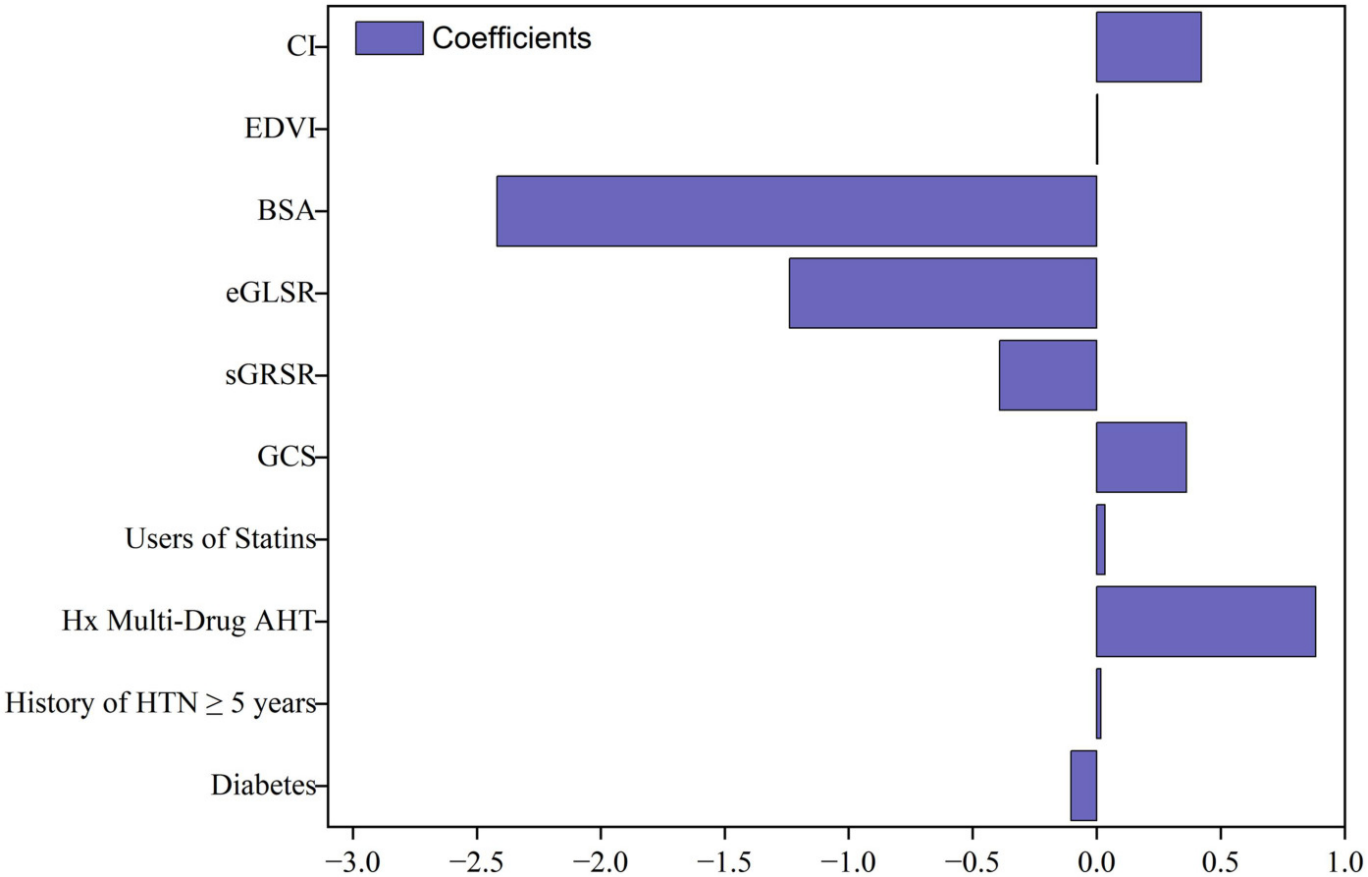
Coefficients of variables selected by LASSO regression (Bar plot of non-zero coefficients at optimal lambda).

**Figure 7:**
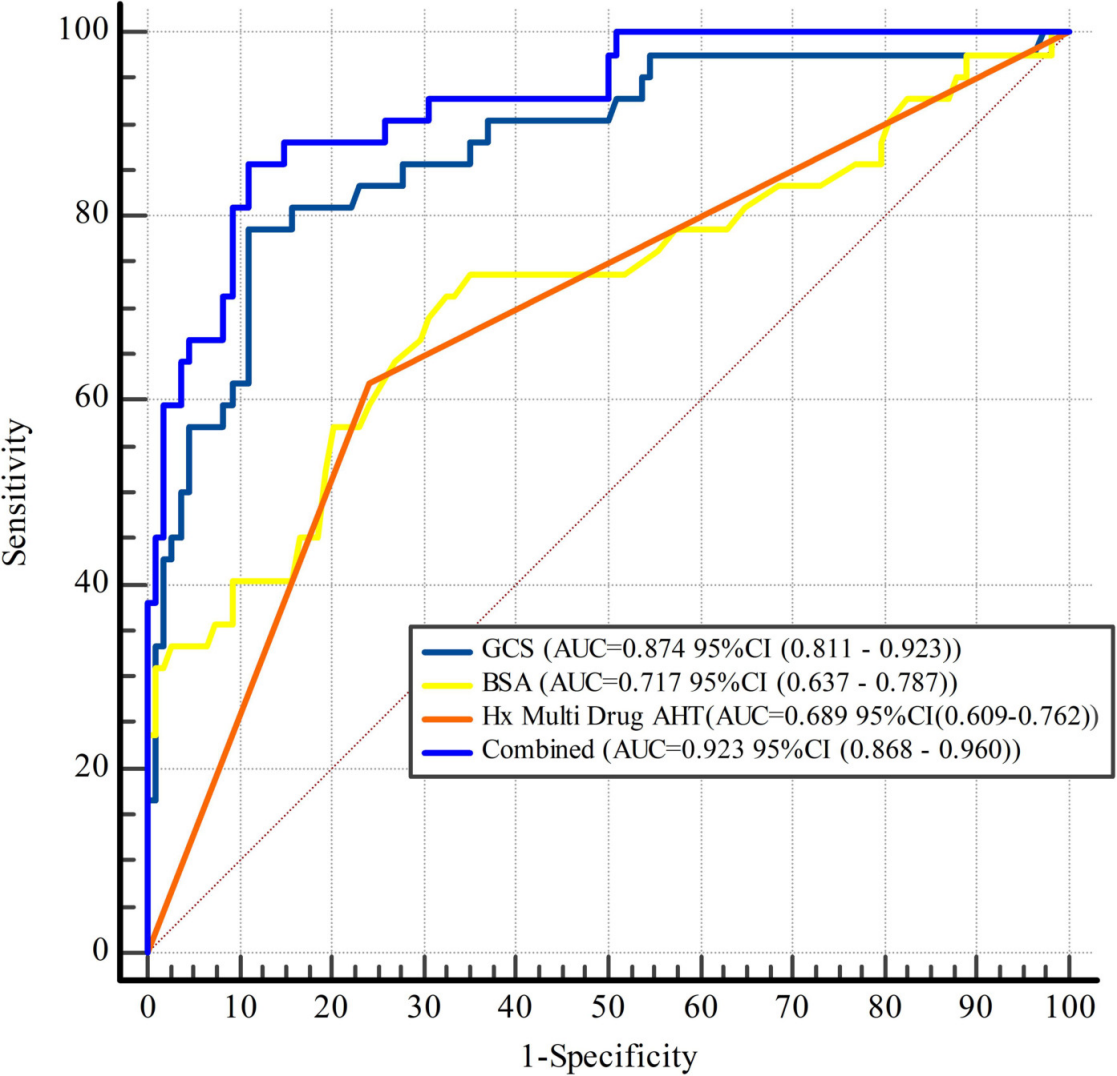
Receiver operating characteristic analysis of risk factors for ventricular arrhythmias in hypertensive patients. The combined indicator includes GCS, BSA, and Hx Multi-Drug AHT. GCS, global circumferential strain; BSA, body surface area; Hx Multi-Drug AHT, History of multi-drug antihypertensive therapy (concurrent use of ≥2 drug classes [e.g., Beta-blocker + Calcium blocker] for ≥3 months; AUC, area under the curve.

LASSO regression was employed for variable selection to construct a logistic prediction model. Figure 5 depicts the coefficients and mean standard error for 10-fold cross-validation. Following LASSO regression, 11 variables with nonzero coefficients were retained, as shown in Figure 6 and Table 4. Collinearity diagnostics revealed variance inflation factors (VIF) ranging from 1.089 to 1.897, indicating no significant multicollinearity among the variables. Multivariate logistic regression analysis demonstrated that only GCS, BSA, and Hx Multi-Drug AHT were independent predictors of VA in HTN patients (all *P* < 0.05), as presented in Table 4. ROC curve analysis (Figure 7) indicated high discriminatory value of GCS for distinguishing VA in HTN patients. Optimal predictive performance was achieved at a GCS cutoff of −17.005% [area under the curve (AUC) = 0.874, 95% CI 0.808–0.941; sensitivity 78.6%, specificity 88.9%]. The combined model integrating GCS, Hx Multi-Drug AHT, and BSA yielded an AUC of 0.923 (95% CI 0.868–0.960) with 85.71% sensitivity and 88.89% specificity.

**Table 4:** Variables selected by LASSO regression and multivariate analysis of VA risk in HTN patients.

Variables	VIF	Multivariable logistic regression		
		*B*	*OR (95% CI)*	*P*
BSA (m^2^)	1.149	−4.786	0.008 (0.001–0.169)	0.02
LVEDVI (mL/m^2^)	1.882	0.006	1.006 (0.950–1.065)	0.842
CI (L/min/m^2^)	1.202	0.882	2.417 (0.881–6.633)	0.087
Diabetes, *n* (%)	1.089	0.461	1.586 (0.311–8.089)	0.579
History of HTN ≥ 5 years	1.092	0.096	1.101 (0.298–4.069)	0.885
Hx Multi-Drug AHT	1.318	−1.704	0.182 (0.046–0.723)	0.015
Users of Statins	1.166	−0.854	0.426 (0.108–1.674)	0.222
GCS (%)	1.773	0.789	2.202 (1.551–3.125)	<0.001
sGRSR (s^−1^)	1.897	−0.724	0.485 (0.153–3.347)	0.523
eGCSR(s^−1^)	1.689	2.157	0.037 (0.012–2.516)	0.262
eGLSR (s^−1^)	1.406	−4.341	0.130 (0.011–1.674)	0.089

HTN, hypertension; VA, ventricular arrhythmia; BSA, body surface area; LVEDVI, left ventricular end-diastolic volume index; CI, cardiac index; Hx Multi-Drug AHT, History of multi-drug antihypertensive therapy (concurrent use of ≥2 drug classes [e.g., Beta-blocker + Calcium blocker] for ≥3 months; GCS, global circumferential strain; sGRSR, global peak systolic radial strain rate; eGCSR, global peak early-diastolic circumferential strain rate; eGLSR, global peak early-diastolic longitudinal strain rate.

### Reproducibility analysis of strain parameters

Table 5 summarizes the intra-observer and interobserver variability of LV strain parameters. The results demonstrated good agreement for all strain parameters except sGLSR. The intra-observer ICCs ranged from 0.862 to 0.927, while interobserver ICCs ranged from 0.751 to 0.900.

**Table 5:** Intra- and inter-observer reproducibility of strain parameters.

Parameters	Intra-observer		Inter-observer	
	*ICC*	*95% CI*	*ICC*	*95% CI*
GRS	0.911	0.823–0.957	0.900	0.802–0.951
GCS	0.927	0.854–0.965	0.864	0.736–0.933
GLS	0.872	0.750–0.937	0.882	0.768–0.942
sGRSR	0.869	0.746–0.935	0.846	0.686–0.918
sGCSR	0.880	0.766–0.941	0.878	0.762–0.940
sGLSR	0.725	0.502–0.859	0.663	0.407–0.824
eGRSR	0.862	0.733–0.932	0.751	0.543–0.873
eGCSR	0.902	0.806–0.952	0.887	0.773–0.944
eGLSR	0.924	0.847–0.963	0.848	0.707–0.924

ICC, intraclass correlation coefficients; GRS, global radial strain; GCS, global circumferential strain; GLS, global longitudinal strain; sGRSR, global peak systolic radial strain rate; sGCSR, global peak systolic circumferential strain rate; sGLSR, global peak systolic longitudinal strain rate; eGRSR, global peak early-diastolic radial strain rate; eGCSR, global peak early-diastolic circumferential strain rate; eGLSR, global peak early-diastolic longitudinal strain rate.

## Discussion

This study utilized CMR-FT to assess LV myocardial mechanics in HTN patients, revealing four key findings. First, HTN patients exhibit early concordant reduction in LV strain parameters—affecting not only GLS but equally GCS and GRS. Second, all diastolic strain rate peaks were significantly lower in HTN patients vs. controls, while systolic strain rates in HTN-LVN showed no statistical difference from controls, indicating diastolic dysfunction may precede systolic impairment. Third, LV strain parameters significantly correlated with blood pressure levels, LVMI, and LVMWT. Fourth, HTN patients with VA exhibited markedly lower myocardial strain parameters than non-VA counterparts, and reduced GCS may serve as an independent risk factor for VA development in this population.

Compared to conventional parameters (LVEF, LVESVI, CI), myocardial strain demonstrates superior sensitivity in detecting early functional remodeling. Our study revealed significantly reduced GLS, GCS, and GRS in HTN patients even when traditional indices remained normal, with these alterations predating LVH development. The underlying mechanisms may involve early microvascular disarray and pathological changes in calcium dysregulation, which precede cardiomyocyte hypertrophy and fibrosis formation, ultimately impairing myocardial deformation capacity (16). Notably, while some studies report preserved or compensatory elevated circumferential/radial strain with initial longitudinal impairment in HTN (17, 18). our cohort demonstrated early concordant reduction across all directional strain components. This pattern suggests global rather than segmental involvement in the HTN remodeling process (19). Even in early-stage HTN, although subendocardial longitudinally-oriented fibers show predominant involvement, interstitial fibrosis extends into mid-myocardial and epicardial layers. Consequently, the myocardial microstructure may already differ significantly from normal myocardium at these initial stages.

In progressive HTN, worsening cardiomyocyte hypertrophy and myocardial fibrosis increase myocardial stiffness while reducing compliance. Concurrently, LVH diminishes capillary density and induces thickening of small coronary artery walls, precipitating microcirculatory dysfunction and myocardial ischemia. These pathological cascades progressively impair myocardial strain (20, 21). Our study demonstrated more pronounced strain impairment in HTN patients with LVH compared to the non-LVH group, indicating that strain deterioration parallels remodeling progression. Consistent with Niu et al.'s findings (22), LVH exerts greater detrimental effects on myocardial strain than diffuse interstitial fibrosis. Our correlation analyses confirmed significant inverse relationships between strain parameters (GRS, GCS, GLS) and both LVMI and LVMWT, establishing that strain reduction escalates with hypertrophy severity. Notably, the negative correlations between these strain parameters (GRS, GCS, GLS) and blood pressure further underscore the clinical imperative for stringent blood pressure control to mitigate mechanical dysfunction.

LV peak diastolic strain rate reflects the maximum myocardial deformation velocity during relaxation. It accurately captures subtle cardiac motion changes independent of tethering effects or global cardiac displacement (23). Our study demonstrated significantly reduced early-diastolic strain rates (eGRSR, eGCSR, and eGLSR) in all HTN patients (regardless of LVH status) compared to controls. Notably, peak systolic strain rates (sGRSR, sGCSR, sGLSR) remained comparable between HTN-LVN and healthy subjects. These findings establish diastolic dysfunction as the earliest functional manifestation of HTN cardiac injury, preceding detectable systolic impairment. Therefore, CMR-FT-derived myocardial strain measurements not only precisely evaluate diastolic dysfunction in HTN patients, but also provide deeper insights into the underlying pathological progression of the disease.

HTN patients are particularly susceptible to VA, which not only represent one of the most frequently observed rhythm disorders in this population (24, 25) but also confer a substantially increased risk of sudden cardiac death (26). Reduced strain in HTN patients has been widely reported (16, 17, 27); however, few studies have investigated strain changes in patients with coexisting VA. Our study demonstrated that compared to HTN patients without VA, those with comorbid VA exhibited significantly reduced strain parameters (GRS, GCS, GLS, sGRSR, sGLSR, eGRSR, eGCSR, eGLSR) alongside altered volumetric parameters—specifically elevated LVEDVI and LVESVI, and decreased LVEF. These findings indicate that arrhythmic comorbidity induces additional LV remodeling, thereby accelerating disease progression in HTN patients. Consequently, accurate assessment and monitoring of cardiac functional impairment in this population are imperative. Notably, VA occurrence showed no association with LVMI or LVMWT in our cohort, contradicting conventional views that prioritize LVH as the primary VA driver (4, 28). Beyond LVH, VA in HTN patients may arise from a combination of factors, including overactivation of the sympathetic nervous and renin-angiotensin-aldosterone systems, electrolyte disturbances and coronary microvascular dysfunction (CMD) (10). Research by Nicola Gaibazzi et al. (29) suggests that reduced myocardial strain may be associated with bystander and silent coronary artery disease. Thus, CMD potentially serves as a key link connecting a spectrum of morphological and functional remodeling (including myocardial hypertrophy, fibrosis, and reduced strain) with VA in HTN patients (30).

Multivariate regression demonstrated that GCS, BSA, and Hx Multi-Drug AHT were independent risk factors for VA in HTN patients, with GCS exhibiting the strongest correlation with VA risk. ROC analysis confirmed the superior diagnostic performance of GCS in distinguishing HTN patients with vs. without VA, and the combination of all three factors further increased the AUC. The LV wall consists of endocardial longitudinal fibers, mid-layer circumferential fibers, and epicardial oblique fibers (31). In HTN patients, early myocardial fibrosis primarily deposits in the subendocardial region, resulting in the most significant impairment in GLS (32). GCS reflects mid-epicardial circumferential motion, and its reduction is associated with mid-layer fibrosis and microcirculatory dysfunction (32, 33). Although GCS is initially less impaired than GLS, it progressively deteriorates with the progression of fibrosis. A significantly reduced GCS may therefore indicate extensive fibrosis, reflecting more severe myocardial structural remodeling and functional impairment, thereby potentially increasing susceptibility to VA. This may explain why reduced GCS demonstrates a stronger association with VA compared to GLS. Previous studies have confirmed that GCS predicts heart failure, myocardial infarction, stroke, and death (34, 35); our findings specifically link reduced GCS to future VA events in HTN patients. Therefore, assessing and monitoring myocardial strain parameters with targeted interventions is critical for HTN prognosis. We confirmed good-to-excellent intra- and inter-observer agreement (ICC 0.75–0.93) for CMR-FT-derived strain parameters, except sGLSR. This auxiliary indicator (vs. GLS as primary parameter) was excluded from our prediction model, thus its reproducibility limitation poses minimal impact.

### Limitations

Several limitations should be acknowledged in this study. First, as a single-center observational study in which all enrolled patients underwent CMR, the findings are subject to selection bias and should not be directly extrapolated to the general HTN population in routine clinical management. In addition, the relatively modest sample size inherent to this retrospective investigation, along with the lack of both external validation in independent cohorts and internal cross-validation, limits the robustness and generalizability of the findings. Second, The absence of longitudinal follow-up data limits prognostic assessment. Future investigations should specifically evaluate the prognostic value of CMR-FT-derived strain parameters in HTN patients, particularly those with concomitant VA, to better define their clinical significance for risk stratification. Third, while CMR-FT demonstrates significant clinical value, its dependency on specialized software and additional post-processing workflows currently limits widespread adoption. The integration of artificial intelligence may streamline strain quantification in future applications. Fourth, while T1 mapping and ECV fraction provide valuable assessment of diffuse fibrosis, technical constraints limited their systematic application in our early cohort. Future prospective studies will incorporate these techniques to examine their interactions with myocardial deformation parameters in VA risk evaluation. Finally, although CMR-FT and echocardiographic strain parameters show good agreement in literature (36, 37), the lack of contemporaneous echocardiographic data in our retrospective cohort precludes direct comparison, requiring verification in future multimodality studies.

## Conclusion

In summary, CMR-FT-derived myocardial strain parameters enable early identification of LV dysfunction in HTN patients. Among these parameters, GCS may serve as a simple and reliable imaging marker to distinguish HTN patients complicated by VA, offering potential as an intervention target for preventing cardiac remodeling and arrhythmia progression in this population.

## Data Availability

The raw data supporting the conclusions of this article will be made available by the authors, without undue reservation.
