# Supplementary material for: Subclinical myocardial dysfunction assessed by cardiac magnetic resonance feature tracking predicts ventricular arrhythmias in early-stage hypertension
Source: Front Cardiovasc Med. 2025 Oct 30;12:1632100. doi: 10.3389/fcvm.2025.1632100 (PMC12611894; doi:10.3389/fcvm.2025.1632100)
Supplement: Supplementary file 1 [file Table1.docx]

Supplementary Table 1. Hyperlipidemia Status Comparison Between HTN Patient Subgroups

|  | **HTN-LVN (79)** | **HTN-LVH (71)** | ***P*** |  | **HTN without VA (108)** | **HTN with VA (42)** | ***P*** |
| --- | --- | --- | --- | --- | --- | --- | --- |
| Hyperlipidemia, n (%) |  |  | 0.207 |  |  |  | 0.387 |
| Positive | 31 (39.2%) | 38 (53.5%) |  |  | 47 (43.5%) | 22 (52.4%) |  |
| Negative | 46 (58.3%) | 31 (45.1%) |  |  | 58 (53.7%) | 20 (47.6%) |  |
| Unverified | 2 (2.5%) | 1 (1.4%) |  |  | 3 (2.8%) | 0 (0%) |  |
